# A randomized controlled study on systematic nursing care based on health empowerment theory and its effect on the self-care and functional abilities of patients with spinal fractures

**DOI:** 10.1186/s13018-023-04317-z

**Published:** 2023-11-01

**Authors:** Hui Li, Lin Gan, Yue Sun, Hui-Ting Yu

**Affiliations:** Department of Orthopedics, The Second Affiliated Hospital of Harbin Medical University, No. 157 of BaoJian Street, NanGang District, Harbin, 150000 China

**Keywords:** Health empowerment theory, Functional ability, Self-care ability, Spinal fractures, Systematic nursing

## Abstract

**Objective:**

In this study, we aimed to explore the effectiveness of systematic nursing care based on health empowerment theory on the self-care and functional abilities of patients with spinal fractures.

**Methods:**

We selected a total of 50 patients with spinal fractures from our hospital and randomly divided them into the control group and the observation group, with 25 patients in each group. Patients in the control group received conventional nursing care, while those in the observation group received systematic nursing care grounded in the health empowerment theory. We recorded and compared the self-care ability, functional ability, knowledge about the condition, and pain scores of patients in the two groups before and after the nursing intervention.

**Results:**

There was no significant difference in the baseline characteristics between the two groups (*P* > 0.05), and there was no significant difference in self-care ability, functional ability, knowledge about the condition, or the visual analog scale (VAS) score between the two groups before treatment (*P* > 0.05). After treatment, outcomes in the observation group in terms of self-care ability, functional ability, and knowledge about the condition were significantly better than those in the control group (*P* < 0.05), while the VAS score in the observation group was significantly lower than that in the control group (*P* < 0.05).

**Conclusion:**

Compared with conventional nursing care, patients with spinal fractures who received systematic nursing inputs based on health empowerment theory reported significant improvements in pain, self-care, functional ability, and knowledge of the condition, and this is an approach that is worthy of promotion in clinical use.

## Introduction

Spinal fracture is a common traumatic disorder that often requires prolonged rehabilitation and treatment, and it is essential for patients to have good self-care and self-decision-making abilities during treatment to help promote their recovery and restore their functional abilities [1]. Patients with chronic diseases and long-term convalescence require long-term self-management and self-care, and these aspects have a crucial impact on patient recovery and quality of life [2]; however, these are often overlooked in modern medicine as the focus is on symptomatic treatment. Therefore, the question of how to empower patients to take charge of their care and make their own decisions has risen to prominence in the medical community.

The traditional nursing model is largely guided by the expertise and skills of healthcare professionals, and patients are often passive receivers of care, unable to actively engage in their treatment and health management [3]. The treatment outcomes and quality of life of patients can be improved with the help of health empowerment theory-based systematic nursing, which is a patient-centered nursing process that emphasizes education and training to improve the self-management ability of patients with respect to their health [4–8]. A spinal fracture is a serious injury and often has considerable physical and psychological consequences [9–13]. The spine is crucial because it links the brain to the rest of the body. As a result, spinal fractures not only affect mobility but can also lead to problems with breathing, excretion, sexual function, and so on. [14]. In addition, spinal fractures can also burden patients psychologically in terms of anxiety and depression, hindering their recovery and quality of life [15]. Research findings [16] have indicated that effective nursing interventions are essential for patients with spinal fractures and can facilitate recovery in many ways.

In light of this, in this study, we compared conventional nursing care with health empowerment-based nursing care for the treatment of patients with spinal fractures and assessed the effects of each model of care on self-care and functional abilities, knowledge of the condition, and pain. Through our findings, we hope to provide theoretical support and practical guidance for promoting the use of systematic nursing interventions based on health empowerment theory in the treatment of patients with spinal fractures.

*Objective*: to explore the effectiveness of systematic nursing care based on health empowerment theory on the self-care and functional abilities of patients with spinal fractures. Hypotheses: Compared with conventional nursing care, patients with spinal fractures who received systematic nursing inputs based on health empowerment theory reported significant improvements in their health status.

## Materials and methods

### Design and setting of study

#### Research participants

In this randomized controlled study, we enrolled a total of 50 patients with spinal fractures from the Orthopedic Department of the Second Affiliated Hospital of Harbin in this study and collected basic sociodemographic information such as gender and age. The patients were randomly assigned to the control group and the observation group, with 25 cases in each group. All patients included in the study had a definite diagnosis of spinal fractures and were treated at our hospital. The study was conducted from January 2022 to June 2022.

### Inclusion and exclusion criteria

1. *Inclusion criteria*: We included patients who fulfilled the following criteria: (1) Patients aged 20–65 years; (2) All patients were diagnosed with fractures of the cervical, thoracic, and lumbar vertebrae, and the time of diagnosis was within 72 h; (3) The degrees of fractures included mild, moderate, or severe; (4) Patients who were willing to participate in this study and sign informed consent; (5) Patients with sufficient cognitive ability to understand the research content and receive appropriate nursing care.
2. *Exclusion criteria*: We excluded the following patients: (1) Excluded patients < 20 years old or > 65 years old; (2) Excluded patients diagnosed with other fracture types; (3) Patients with fractures longer than 72 h were excluded; (4) Exclude patients with other serious diseases of the heart, lung, liver, kidney and other systems; (5) Exclude patients with cognitive impairment or mental illness; (6) Exclude patients with other bone diseases or osteoporosis; (7) Excluding refusal to sign the informed consent or inability to fully cooperate with the researcher (patient and family) for various reasons.

A total of 50 patients admitted to hospital during this period were selected and numbered 1–50. The observation group was numbered in odd numbers, and the control group was numbered in even numbers.

### Nursing intervention

1. Patients in the control group received conventional nursing care, which included the following interventions: explaining the condition and measures to ensure patient comfort, regular monitoring of patient condition and pain, and timely administration of prescribed medications and physical therapy.
2. Patients in the observation group received systematic nursing care inputs based on the health empowerment theory, which included a personalized systematic nursing plan formulated according to the individual needs of patients and the health empowerment theory. The specific measures were as follows:
  1. *Pain management*: Patients underwent an assessment of their pain levels, and based on the results, various pain management strategies such as the use of analgesic drugs, physical therapy, and the like were adopted. Meanwhile, patients were given advice on practicing self-management techniques for dealing with pain such as deep breathing, relaxation, and so on [5].
  2. *Functional recovery*: A customized rehabilitation program, including appropriate exercise and adjustment of body posture, was developed based on the type and degree of fracture. Additionally, patients were educated on self-help skills for rehabilitation, such as precautions in performing daily activities and exercise methods, and so on to promote functional recovery.
  3. *Knowledge education*: We offered individualized education programs for patients to improve their understanding of fractures, increase their awareness of their condition and guided them on how to prevent and respond to possible complications as well [6].
  4. *Psychological support*: As per the psychological state of the patients, we offered positive psychological interventions such as emotional support, positive psychological training and the like to relieve their psychological stress, and improve their self-esteem and compliance with treatment [7].
  5. *Monitoring of the condition*: We assessed and monitored patient condition on a regular basis, and initiated the corresponding adjustments in treatment and interventions. We identified and treated complications in time. At the same time, we also monitored the recovery status through regular follow-ups to ensure the best outcomes for recovery.
  6. *Sleep management*: We implemented appropriate measures to manage the possible sleep problems of patients, such as enhancing the sleep environment, modifying the sleep position, using sedatives, and so on so as to improve the quality of sleep and comfort.
  7. *Diet management*: We formulated an appropriate diet plan and targeted nutritional guidance including controlling the amount of food intake and the proportion of nutrients as per the medical and physical condition of the patients to improve their nutritional status and immunity.
  8. *Prevention of accidental injury*: We addressed reducing the risk of accidental injury by educating patients about preventing accidental injury, including how to avoid falls, how to use walkers correctly, and how to avoid traffic accidents [8].
  9. *Social support*: We facilitated the provision of necessary social support and assistance for patients, including family care, rehabilitation facilities and social resources, among other aspects so as to improve the quality of life and rehabilitation outcome of patients.

### Observation indicators

1. *Level of self-care ability*: We used the Self-Management Assessment Scale [9] to evaluate the self-care ability of patients both before and after treatment. The scale has a total of 20 items and a score range of 20–100 points to assess self-care, self-monitoring, self-regulation, and so on. The higher the scores, the better the self-care ability.
2. *Level of functional ability*: We evaluated the functional ability of patients before and after treatment using the Activities of Daily Living (ADL)scale [10]. The ADL scale contains a total of 6 items, and the response for each item is “completely independent” (scored as 2), “partially dependent” (scored as 1), or “completely dependent” (scored as 0), with a total score range of 0–12 points. The higher the scores, the better the functional ability. ADL Scale, developed by Lawton and Brody in the United States in 1969. It is mainly used to assess the daily living ability of the subjects. The research proves that it has high retest reliability and validity, good internal consistency, and is a reliable and effective evaluation scale for activities of daily living ability.

Self-management ability scale: The Cronbach coefficient of this scale is 0.920, and the internal consistency coefficient of each part is 0.718–0.909, which has good reliability and validity.

(3) *Extent of awareness about the condition*: We used the “spinal fracture general information questionnaire” for the survey of patients to understand the extent of their knowledge about spinal fracture before and after treatment [11]. The scale includes information pertaining to causes, symptoms, treatment, and other aspects, with a score range of 0–100 points. The higher the scores, the better the awareness of the condition.
(4) *Level of pain*: We used a visual analog scale (VAS) [12] before and after treatment to evaluate the pain level of the patients. The scores on the VAS scale range from 0 to10 points. The higher the score, the more severe the pain.

### Statistical analysis

We used the SPSS 22.0 software for statistical analysis. For measurement data, we used the mean and standard deviation to describe its distribution in this study, and the *t*-test or analysis of variance for statistical analysis to compare data between the two groups. For enumeration data, we used frequency and percentage to describe its distribution, and the Chi-square test or Fisher’s exact test was adopted for statistical analysis to compare the data between the two groups. *P* < 0.05 indicated that the difference was statistically significant.

## Results

### Comparison of basic data of the two groups of patients

There were 13 males and 12 females among the 25 patients in the observation group. The age range was 20–65 years, with a mean age of (40.7 ± 5.2) years. Based on the type of fracture, there were 4 cases of thoracic vertebral fracture, 16 cases of lumbar vertebral fracture, and 5 cases of cervical vertebral fracture. The proportion based on the degree of severity of the fracture was: 6 cases of mild fracture, 10 cases of moderate fracture, and 9 cases of severe fracture. As per education level, there were 21 patients with a high school degree or less, and 4 patients with a junior college degree or higher.

In the 25 patients in the control group, there were 14 males and 11 females. The age range was 20–65 years, with a mean age of (39.8 ± 5.5) years. Based on the type of fracture, there were 5 cases of thoracic vertebral fracture, 15 cases of lumbar vertebral fracture, and 5 cases of cervical vertebral fracture. As per the severity of the fracture, 5 cases had a mild fracture, 11 cases had a moderate fracture, and 9 cases had a severe fracture. Regarding the education level of the patient, 20 patients had a high school degree or less, and 5 patients had a junior college degree or higher.

Overall, the gender, age, type of fracture, severity of the fracture, level of education of the patient, and other information of the patients were comparable between the two groups, and the difference was not statistically significant (*P* > 0.05) (Table 1).

**Table 1:** Comparison of basic information between the two groups

Items	Control group (*n* = 25)	Observation group (*n* = 25)	*t*/*x*^2^	*P*
Gender			0.080	0.776
Male	13	14		
Female	12	11		
Age (years)	40.7 ± 5.2	39.8 ± 5.5	0.619	0.538
Type of fracture			0.0	1.0
Thoracic vertebra	4	5		
Lumbar vertebra	16	15		
Cervical vertebra	5	5		
Severity of fracture			0.0	1.0
Mild	6	5		
Moderate	10	11		
Severe	9	9		
Educational level			0.0	1.0
High school and below	21	20		
Junior college or above	4	5		

### Comparison of self-care ability of patients in the two groups

There was no significant difference in self-care ability between patients in the two groups before treatment (*P* > 0.05). After treatment, we found that the self-care ability scores of patients in the observation group were significantly higher than those of the control group (*P* < 0.05) (Table 2).

**Table 2:** Comparison of self-care ability levels between the two groups

Group	Self-care ability levels (scores)	
	Before treatment	After treatment
Control group (*n* = 25)	46.2 ± 7.6	63.4 ± 6.7
Observation group (*n* = 25)	45.8 ± 8.3	76.5 ± 8.1
*t*	0.177	6.231
*P*	0.859	1.11141E-07

### Comparison of functional ability of patients in the two groups

Before treatment, there was no significant difference in the functional ability scores of patients in the two groups (*P* > 0.05), but after treatment, the functional ability scores of patients in the observation group were significantly higher than those of the control group (*P* < 0.05) (Table 3).

**Table 3:** Comparison of levels of functional ability between the two groups

Group	Functional ability levels (scores)	
	Before treatment	After treatment
Control group (*n* = 25)	3.1 ± 1.3	7.3 ± 1.6
Observation group (*n* = 25)	3.2 ± 1.4	8.5 ± 1.5
*t*	0.261	2.735
*P*	0.794	0.008

### Comparison of knowledge of the condition between the two groups

There was no significant difference in the level of awareness of the condition among patients between the two groups before treatment, (*P* > 0.05). After treatment, patients in the observation group had a significantly higher level of awareness about the condition when compared with the control group (*P* < 0.05) (Table 4).

**Table 4:** Comparison of knowledge of the condition between the two groups

Group	Knowledge of the condition (scores)	
	Before treatment	After treatment
Control group (*n* = 25)	40.6 ± 8.2	61.8 ± 8.9
Observation group (*n* = 25)	41.3 ± 7.5	78.4 ± 6.7
*t*	0.315	7.450
*P*	0.754	1.50377E-09

### Comparison of pain status (VAS scores) between the two groups

There was no significant difference in the pre-treatment VAS scores between the two groups (*P* > 0.05). After treatment, we found that the VAS scores of patients in the observation group were significantly lower than those in the control group (*P* < 0.05) (Table 5).

**Table 5:** Comparison of pain levels (VAS scores) between the two groups

Group	VAS score (scores)	
	Before treatment	After treatment
Control group (*n* = 25)	7.2 ± 1.4	4.5 ± 1.2
Observation group (*n* = 25)	7.3 ± 1.5	2.9 ± 1.4
*t*	0.243	4.338
*P*	0.808	0.001

## Discussion

Traditional nursing care was the mainstay of previous clinical nursing interventions for patients with spinal fractures. Despite its potential benefits, this model of nursing care has struggled for a long time to meet the expectations of both clinicians and patients. Based on an analysis of my observations based on personal experience and related researches [17, 18], I have summarized several shortcomings in conventional nursing:

(1) Unsatisfactory recovery of limb function: While conventional nursing ensures that the basic needs of patients are met, there is not enough attention and effective intervention for the recovery of limb function in patients with spinal fractures, resulting in some patients being unable to fully recovery use of their limbs, thus affecting the quality of life; (2) Insufficient pain control: Patients with spinal fractures often experience severe pain during treatment, and conventional nursing has limited interventions for pain control, often resorting to medications or other specific therapies; (3) Inadequate nutritional support: Patients with spinal fractures need a lot of energy and nutrition during treatment and recovery, and there is a lack of awareness and application of nutritional support in conventional nursing which needs to be addressed; (4) Insufficient attention to psychological aspects: Patients with spinal fractures often face severe psychological stress and negative emotions such as depression and anxiety, during the process of treatment and recovery, and more attention is required to be paid to mental health care in conventional nursing. Therefore, finding a more effective nursing intervention model has become a major clinical challenge for orthopedics [19].

In traditional nursing, nurses divide their work according to specific nursing tasks such as injection, infusion and dispensing of medicine. The quality nursing service carries out the responsibility system, and the nurses carry out the overall responsibility system for the patients through the division of labor in the form of patient package, including basic nursing, treatment and drug administration, rehabilitation guidance, health education, psychological nursing and other nursing work [20].

In terms of the connotation of nursing, the traditional nursing model makes each nurse's care for patients fragmented and focuses on treatment, ignoring the overall care of patients, while high-quality nursing services provide patients with comprehensive, whole-process, seamless overall care, and improve the quality of nursing services [20].

Health empowerment theory-based systematic nursing is a nursing method that addresses individualized needs of patients and enhances their self-management capacity to empower patients, promote recovery, and prevent disease recurrence [19]. This patient-centered nursing approach emphasizes an equal partnership between caregivers and patients. It enables patients to learn to take responsibility for their own health and wellbeing and make better choices for themselves via education, advice, support, and encouragement [20–22].

In this study, the patients in the observation group received systematic nursing inputs for spinal fractures based on the health empowerment philosophy. As compared with the control group receiving conventional nursing intervention, the patients in the observation group showed significant improvements in their self-care and functional abilities, awareness of the condition, and VAS score after the systematic nursing intervention. The results of this study showed that health empowerment theory-based systematic nursing significantly enhanced awareness of the condition, self-care and functional ability, and significantly relieved pain in patients with spinal fractures, thereby promoting the recovery of health.

Probable reasons for our results are as follows: (1) Health empowerment theory-based systematic nursing is based on evaluation and analysis of the individual needs of patients to formulate a personalized nursing plan that addresses the unique situation of each patient to achieve more accurate and effective care; (2) Health empowerment theory-based systematic nursing emphasizes patient involvement and participation, which can improve self-decision-making and self-efficacy by encouraging and supporting patients to participate in self-management and nursing processes, thereby stimulating their enthusiasm; (3) Health empowerment theory-based systematic nursing focuses on prevention, which not only focuses on the current health status of patients, but also pays attention to the prevention of diseases. It helps patients learn correct self-care and preventive measures through education and guidance, so as to reduce the risk of disease-related adverse reactions, and then, promote rapid recovery; (4) Health empowerment theory-based systematic nursing emphasizes comprehensive nursing care, which includes inputs to address physical, psychological, social, and other aspects of patient care to comprehensively improve the health of patients.

While this study has some strengths, it also has some limitations, including (but not limited to) the following: (1) the sample size is small and the duration of follow-up is short; (2) there is a lack of specifics regarding the nursing intervention program; (3) there is no evaluation of the level of satisfaction with the interventions; (4) there may be some biases in the selection of participants due to differences in age, gender, education level, and other factors. Overall, this study has some useful findings, and the shortcomings must be addressed in future research in order to increase the reliability and generalizability of the results. To sum up, although this study has achieved some positive results, there are still shortcomings in the study itself. Therefore, future studies should be improved accordingly to improve the reliability and generalization of the study.

## Conclusion

In conclusion, compared with conventional nursing care, systematic nursing inputs grounded in health empowerment theory were more effective than conventional nursing care in alleviating pain in patients with spinal fractures, improving self-care and functional abilities, and enhancing awareness of their condition, suggesting that this model of care merits greater promotion and clinical use.

## Implications for nursing practice

Systematic nursing based on health empowerment theory could significant improve patients’ status in pain, self-care, functional ability, and knowledge of the condition.

## Data Availability

The data used to support the findings of this study are available from the corresponding author upon request.

## References

[1] Tang C, Jiang T, Chen X (2022). Application effect of diversified health education combined with fast-track surgery nursing in spinal fracture patients. J Clin Nurs Pract.

[2] Hamilton K, Josiah DT, Tierney M, Brooks N (2019). Surgical practice in traumatic spinal fracture treatment with regard to the subaxial cervical injury classification and severity and the thoracolumbar injury classification and severity systems: a review of 58 patients at the University of Wisconsin. World Neurosurg.

[3] Fei Y, Li X (2021). Comparison of the effects of operating room systematic care and usual care in reducing intraoperative stress injury in patients with spinal fracture with spinal cord injury. Diet Health.

[4] Wang H, Tan X, Xie Y (2021). A comparative study of clinical education pathway and systematic health education in postoperative rehabilitation care after spinal fracture. Med Diet Health.

[5] Alsoof D, Anderson G, McDonald CL, Basques B, Kuris E, Daniels AH (2022). Diagnosis and management of vertebral compression fracture. Am J Med.

[6] Bork H, Simmel S, Böhle E (2018). Rehabilitation nach traumatischen Frakturen der Brust- und Lendenwirbelsäule [rehabilitation after traumatic fracture of thoracic and lumbar spine]. Z Orthop Unfall.

[7] Wang J (2019). Exploring the application value of systematic health education in the rehabilitation care of spinal fracture. Gems Health.

[8] Park MS, Moon SH, Yang JH, Lee HM (2013). Neurologic recovery according to the spinal fracture patterns by Denis classification. Yonsei Med J.

[9] Sandal LF, Bach K, Øverås CK (2021). Effectiveness of app-delivered, tailored self-management support for adults with lower back pain-related disability: a selfBACK randomized clinical trial. JAMA Intern Med.

[10] Tago M, Katsuki NE, Nakatani E (2022). Correction: external validation of a new predictive model for falls among inpatients using the official Japanese ADL scale, Bedriddenness ranks: a double-centered prospective cohort study. BMC Geriatr.

[11] Amer M, Noor S, Kashif SM, Nazir SUR, Ghazanfar T, Yousaf S (2021). Evaluation of disease related knowledge in patients of osteoporosis: an observational study. Altern Ther Health Med.

[12] Thong ISK, Jensen MP, Miró J, Tan G (2018). The validity of pain intensity measures: what do the NRS, VAS, VRS, and FPS-R measure?. Scand J Pain.

[13] Yu J, Hu X (2018). Effect of systematic nursing on negative emotions and functional rehabilitation in patients with spinal fracture surgery. Chin Foreign Med Res.

[14] Jiang B, Zhu R, Cao Q, Pan H (2014). Severe thoracic spinal fracture-dislocation without neurological symptoms and costal fractures: a case report and review of the literature. J Med Case Rep.

[15] Kreinest M, Rillig J, Küffer M, Grützner PA, Tinelli M, Matschke S (2021). Comparison of pedicle screw misplacement following open vs. percutaneous dorsal instrumentation after traumatic spinal fracture. Eur J Trauma Emerg Surg.

[16] Cong Q (2022). The application of intraoperative systematic nursing coordination in patients with spinal fracture and spinal cord injury. Chin J Trauma Disabil Med.

[17] Lu Q (2022). The ison of operating room systematic care and routine care in reducing spinal fracture and intraoperative stress injury in patients with spinal cord injury. Chin Baby.

[18] Zilbermints V, Hershkovitz Y, Peleg K (2021). Spinal cord injury in the setting of traumatic thoracolumbar fracture is not reliably associated with increased risk of associated intra-abdominal injury following blunt trauma: an analysis of a National Trauma Registry database. Chin J Traumatol.

[19] Wang X, Xu X (2022). Effect of systematic care based on health empowerment theory on self-efficacy and complications in elderly patients with fragility fractures. Modern J Integr Tradit Chin Western Med.

[20] Kang Y, Xing L, Zhang Y (2020). Effects of video teaching combined with empowerment theory on rehabilitation training in elderly patients undergoing total knee arthroplasty. Chin J Pract Nurs.

[21] Wang C, Zhao Y, Li R (2018). Application of health empowerment theory in postoperative rehabilitation management of elderly patients with femoral fracture. Modern Nurs.

[22] Dong Y, Wang J (2020). Application value of health empowerment theory in nursing elderly patients with brittle fracture. J Bengbu Med Coll.

